# Concordance of left ventricular volumes and function measurements between two human readers, a fully automated AI algorithm, and the 3D heart model

**DOI:** 10.3389/fcvm.2024.1400333

**Published:** 2024-07-16

**Authors:** Peder L. Myhre, Nicola Gaibazzi, Domenico Tuttolomondo, Daniele Sartorio, Pietro Tito Ugolotti, Marco Covani, Alberto Bettella, Sergio Suma

**Affiliations:** ^1^ Department of Cardiology, Akershus University Hospital, Lørenskog, Norway; ^2^ K.G. Jebsen Center of Cardiac Biomarkers, University of Oslo, Oslo, Norway; ^3^ Cardiology Department, University Hospital of Parma, Parma, Italy

**Keywords:** artificial intelligence, echocardiography, ejection fraction, global longitudinal strain, 3D echocardiography

## Abstract

**Background:**

Echocardiography is essential in cardiovascular medicine for screening, diagnosis, and monitoring. Artificial intelligence (AI) has the potential to improve echocardiography by reducing variability and analysis time. While 3D echocardiography is becoming more accurate, 2D imaging still dominates clinical care. We aimed to evaluate agreement in measures of left ventricular (LV) volumes and function between human readers, a fully automated AI 2D algorithm, and the 3D Heart Model.

**Methods:**

A retrospective analysis was conducted on 109 patients who underwent 2D and 3D transthoracic echocardiography. LV end-diastolic and end-systolic volumes (LVEDV, LVESV) and ejection fraction (LVEF) were measured by two operators, a commercially available AI algorithm (US2ai), and the 3D Heart Model. Global longitudinal strain (GLS) was measured by the integrated semi-automated software and the AI algorithm. Outcomes included measures of agreement [bias, limit of agreement and Pearson's correlation (R)]

**Results:**

For LV volume measurements, the AI algorithm was strongly correlated with the average of the human operators (*r* = 0.89 for LVEDV and *r* = 0.92 for LVESV), which was higher than between the operators (*r* = 0.74 and *r* = 0.84, respectively, *p* < 0.01). The same trend was seen for measures of reliability with respect to LVEDV, but not LVESV. AI demonstrated comparable performance to human operators in measuring LVEF, while the 3D Heart Model had a weaker correlation and reliability compared with human operators and AI measurements. The correlation between human operators and AI for GLS was only moderate.

**Conclusion:**

This study demonstrates AI-based echocardiography as a promising tool for accurately assessing LV volumes and LVEF in clinical practice. AI-based measures demonstrated a significantly lower inter-operator variability, thereby improving the consistency and reliability of these assessments. Moreover, AI may prove particularly effective for conducting retrospective bulk analyses, offering a valuable tool for comprehensive evaluations of past data.

## Introduction

Echocardiography holds a pivotal role in multiple aspects of cardiovascular medicine, encompassing screening, prevention (e.g., in patients undergoing cardiotoxic cancer treatments), diagnosis, risk stratification or monitoring for structural and functional abnormalities (1–3). The integration of artificial intelligence (AI) has already proven its value in various cardiac imaging modalities and has the potential to significantly enhance or simplify echocardiography as well. By eliminating intra-operator and inter-operator variability, AI may minimize the need for extensive training programs for operators, or AI can expedite the analysis time required to interpret collected images, leading to more efficient diagnosis and decision-making processes (4–6).

Although 3D echocardiography is becoming increasingly easy and accurate, 2D imaging is still the work horse of everyday echocardiography primarily due to technical limitations and availability of 3D echocardiography. However, automatic measurements of 3D datasets using near real-time machine learning techniques have revolutionized the clinical applicability of 3D echocardiography, especially in quantifying chamber volumes and ejection fraction. Nonetheless, these methods are often vendor-specific and primarily available in top academic centers.

Automated AI algorithms that are capable of accurately analyzing standard 2D echocardiography are highly desirable, both for routine clinical practice and for retrospective automated analysis of the large amounts of echocardiograms stored in electronic archives worldwide. By enabling automated analysis, valuable and unexpected longitudinal variations in key parameters and their trajectories could be revealed, reducing the necessity for time-consuming assessments by expert human readers, opening new roads for retrospective analyses of data. However, ensuring that AI-based automatic measurements perform at least as good as manual readings remains a critical requirement.

Our aim was to assess the agreement, correlation, and reliability of measurements performed by (a) a fully automated commercially available AI-algorithm (Us2ai) on 2D images, (b) the Heart Model 3D (HM3D) system and (3) human expert readers.

## Methods

This was a retrospective analysis of 109 consecutive subjects who underwent transthoracic echocardiography at the cardiology echo lab of the University Hospital of Parma, a tertiary care center, between November 1 and December 1, 2022. The study protocol was approved by the institutional review board.

### Transthoracic image analyses

All patients underwent a resting transthoracic echocardiogram according to international guidelines (7). 2D and 3D ultrasound imaging was performed using an EPIQ machine and ×5 transducer by Philips Healthcare. The HM3D images were obtained by employing wide-angle acquisition in “full-volume” mode, optimizing the frame rate by minimizing sector depth and width. Images were later analyzed off-line by two experienced operators who were blinded to each other and clinical data. In particular, experienced operator had EACVI transthoracic echocardiography certification or an echocardiography experience of more than 10 years. Left ventricular end-diastolic volumes (LVEDV), left ventricular end-systolic volumes (LVESV) and left ventricular ejection fraction (LVEF) were calculated using the modified Simpson's rule according to the 2015 American Society of Echocardiography (ASE)/European Association of Cardiovascular Imaging (EACVI) guidelines for cardiac chamber quantification (7). Peak R wave and end of T wave on ECG were used to identify end-diastole and end-systole, respectively, for manual measurements (each reader used these same criteria, also when repeating measurements for intra- inter-observer variability), while 2D AI and 3D heart model systems identify end-diastole and end-systole with proprietary methods. We selected only cineloops not comprising arrhythmias from analyses, to avoid potential confounders. Global longitudinal strain (GLS) was calculated as the average Legrangian strain from the apical 4-chamber (A4C), apical 3-chamber (A3C) and apical 2-chamber (A2C) views using the conventional software *Autostrain* (Philips Healthcare), which is semi-automated (i.e., operators acquiring the images adjust the endocardial border tracings if needed) (8).

The semi-automated 3DHM algorithm was used to determine 3D measures of LVEDV, LVESV with the aim to calculate only LVEF, since 3D volumes were deemed not comparable to 2D volumes.

Briefly, 3D datasets were acquired in a single beat during a breath hold lasting a few seconds, ensuring optimal temporal and spatial resolution. The volumetric datasets were immediately evaluated on-board using the DHM software (Heart Model, Philips Healthcare), which automatically identifies LV endo- and epicardial borders at end-diastole and LA borders at end-systole, allowing prompt quantification of the volumes of these chambers In our study, 3DE images were analyzed using the default settings of the boundary detection sliders (end-diastolic default position = 60/60; end-systolic default position = 30/30).

The fully-automated 2D AI-based analyses were performed by the commercially available algorithm from Us2ai (Us2ai, Singapore, Singapore), which automatically calculated LV volumes, LVEF and GLS without any manual correction. The algorithm is based on a deep learning workflow, as previously described for 2D videos and GLS (9, 10). In brief, the AI algorithm classifies the 2D video clips into either A4C, A3C or A2C view and automatically excludes low-quality images. Then, automated contouring of the endocardial border for every frame from the A4C, A3C and A2C views are performed by a convoluted neural network (CNN) model. Automated identification of the end-diastolic and the end-systolic frames based upon video-level volume curves with confirmation by an accompanying electrocardiogram, if available. The strain module uses the annotated and endocardium-traced video clips of LV produced in the conventional 2D echo module to measure the circumferential lengths of a traced endocardium for each frame and are projected as drift corrected strain curves based on the cardiac cycle identified by video level volume curves.

### Statistical methods

Unless otherwise specified, data are presented as mean +/− SD or *n* (%). Group comparisons were performed using the Student's *t*-test or a Mann–Whitney *U* test for continuous data and categorical data were compared with chi-squared (*χ*^2^) test. Bland-Altman plots were utilized to assess methodological agreement, including bias (difference in mean measurement) and 95 percent limits of agreement (LoA, mean of the two measurements ± 1.96 × SD) between the methods. Paired *t*-tests were conducted to determine the significance of the biases. Measurement variability was expressed as the mean absolute difference (MAD) between corresponding pairs of repeated measurements within each patient throughout the study group. Correlations were assessed using the Pearson coefficient (r). Reliability was evaluated using the interclass correlation coefficient, which considers the average of K to determine the degree of reliability among the different methods.

*P*-value < 0.05 was considered statistically significant.

## Results

The human operators and the AI algorithm successfully analyzed all 109 (100%) 2D echocardiographic studies included in our study, while the 3DHM algorithm was able to analyze 99 of the studies (89%). The clinical characteristics of the study population are presented in Table 1.

**Table 1:** clinical characteristics of the studied population.

Age, years (mean ± SD)	56 ± 15
Females (*n*, %)	77 (71%)
Family history of CVD (*n*, %)	59 (54%)
Cigarette smoking history (*n*, %)	42 (39%)
Arterial hypertension	45 (41%)
Diabetes mellitus (*n*, %)	17 (16%)
Obesity (*n*, %)	31 (28%)
Dyslipidemia (*n*, %)	45 (41%)
Biplane LVEF^a^ (mean ± SD)	63 ± 8

CVD, cardiovascular disease; LVEF, left ventricle ejection fraction.

^a^ The average of two human operators.

Absolute mean values for each measurement performed with different methods (LVEDV, LVESV, LVEF, GLS) are presented in Table 2.

**Table 2:** LVEDV, LVESV, LVEF and GLS mean values (standard deviation) for human operators, AI and 3DHM.

	Human operators and Autostrain	AI	3DHM
LVEDV (ml)	93.01 (28.34)	90.21 (28.25)	129.21 (3,917)
LVESV (ml)	39.60 (17.61)	33.81 (16.54)	53.01 (21.91)
LVEF (%)	57.63 (6.65)	63.31 (7.73)	59.40 (6.11)
GLS (%)	−18.25 (3.35)	−22.14 (3.55)	

**Figure 1:**
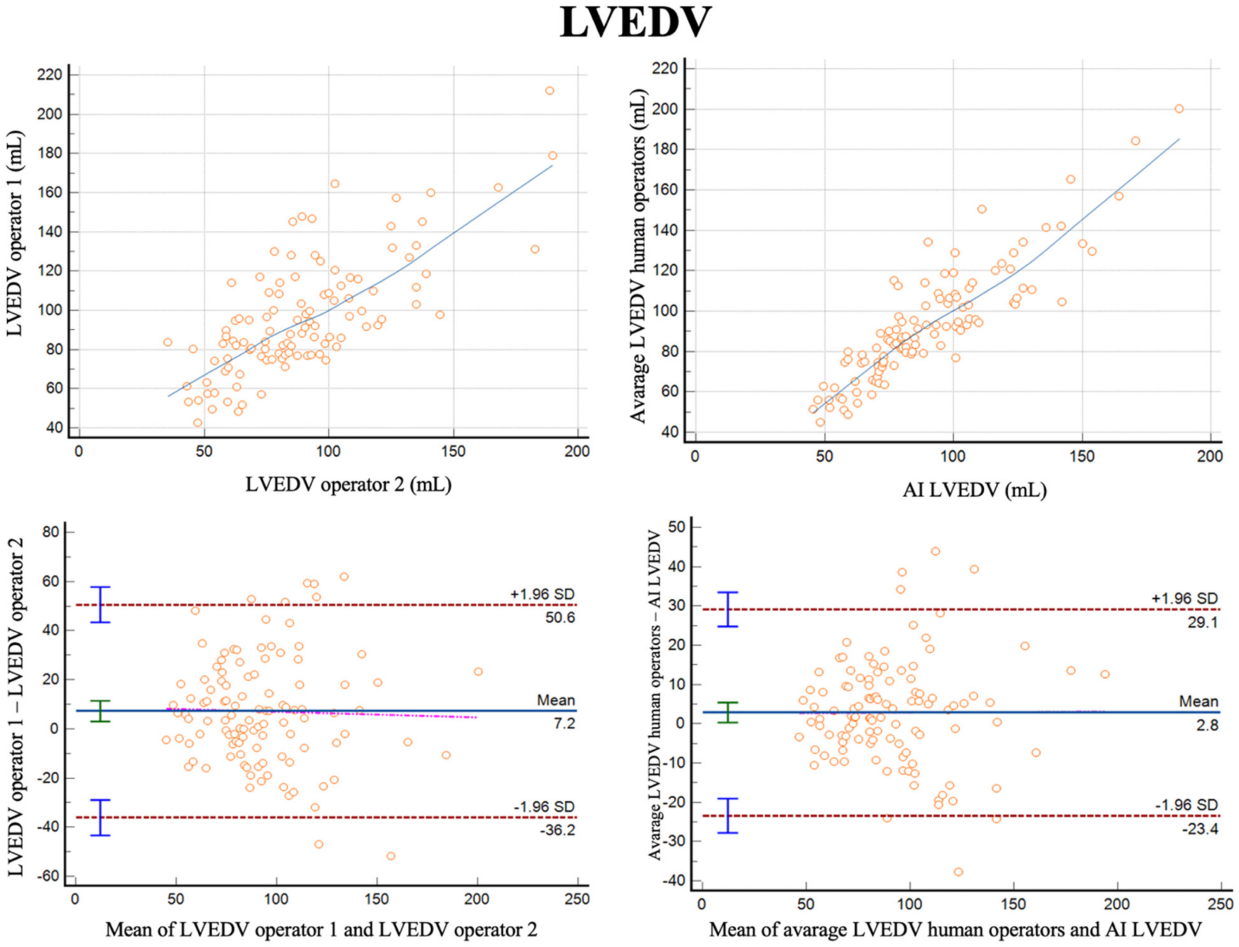
Correlation plots and bland-altman plots of left ventricular end diastolic volume (LVEDV) measures between two human operators (left) and between AI-based measures and the average between the human operators (right).

For measurements of LVEDV, the correlation between the two operators was *r* = 0.74 (95% CI 0.64–0.81, *p* < 0.001), with a reliability of k = 0.85 (Figure 1; Table 3). The average bias between the operators was 7.2 ml (LoA ± 43.4 ml). Comparing the average of the operators with AI, the correlation was *r* = 0.89 (0.84–0.92, *p* < 0.001), with a reliability of k = 0.94. The average bias was 2.8 ml (LoA ± 26.3 ml).

**Table 3:** Bias, correlation and reliability for measures of left ventricular volume, ejection fraction and global longitudinal strain by human operators, 2D AI algorithm and 3D heart model.

	Agreement Bland and Altman		Correlation	Reliability (intraclass correlation coefficient)	
	BIAS	LOA 1.96 SD	Pearson's *r*	Single rating	Average of K
LVEDV, ml					
Between 2 operators	7.2	43.4	0.736 (0.635–0.811)	0.735 (0.636–0.811)	0.847 (0.777–0.896)
Mean Operators vs. 2D AI	2.8	26.3	0.888 (0.840–0.922)	0.888 (0.840–0.922)	0.941 (0.913–0.959)
Same reader 2 measures	−0.4	27.9	0.893 (0.847–0.925)	0.894 (0.848–0.926)	0.944 (0.918–0.962)
-LVESV, ml					
Between 2 operators	5.7	20.8	0.840 (0.774–0.888)	0.834 (0.766–0.883)	0.910 (0.868–0.938)
Mean Operators vs. 2D AI	−11.9	37.6	0.924 (0.891–0.948)	0.428 (0.262–0.570)	0.600 (0.415–0.726)
Same reader 2 measures	−1.1	15.9	0.904 (0.862–0.933)	0.899 (0.856–0.930)	0.947 (0.922–0.964)
LVEF, %					
Between 2 operators	−2.4	11.5	0.684 (0.569–0.773)	0.683 (0.568–0.771)	0.812 (0.725–0.871)
Mean Operators vs. 2D AI	−5.2	11.2	0.697 (0.586–0.783)	0.692 (0.579–0.778)	0.818 (0.734–0.875)
Same reader 2 measures	1	11	0.745 (0.648–0.818)	0.737 (0.637–0.812)	0.849 (0.778–0.896)
Mean Operators vs. 3D HM	−0.6	13.4	0.623 (0.485–0.731)	0.608 (0.468–0.719)	0.756 (0.637–0.836)
3D HM vs. 2D AI	−4.4	11.4	0.689 (0.542–0.794)	0.668 (0.516–0.779)	0.801 (0.681–0.876)
Fully automated LV GLS, %					
Autostrain1 2D vs. AI strain	4	6.3	0.552 (0.401–0.674)	0.552 (0.402–0.673)	0.711 (0.573–0.804)

Correlation and reliability data and (95% confidence interval).

**Figure 2:**
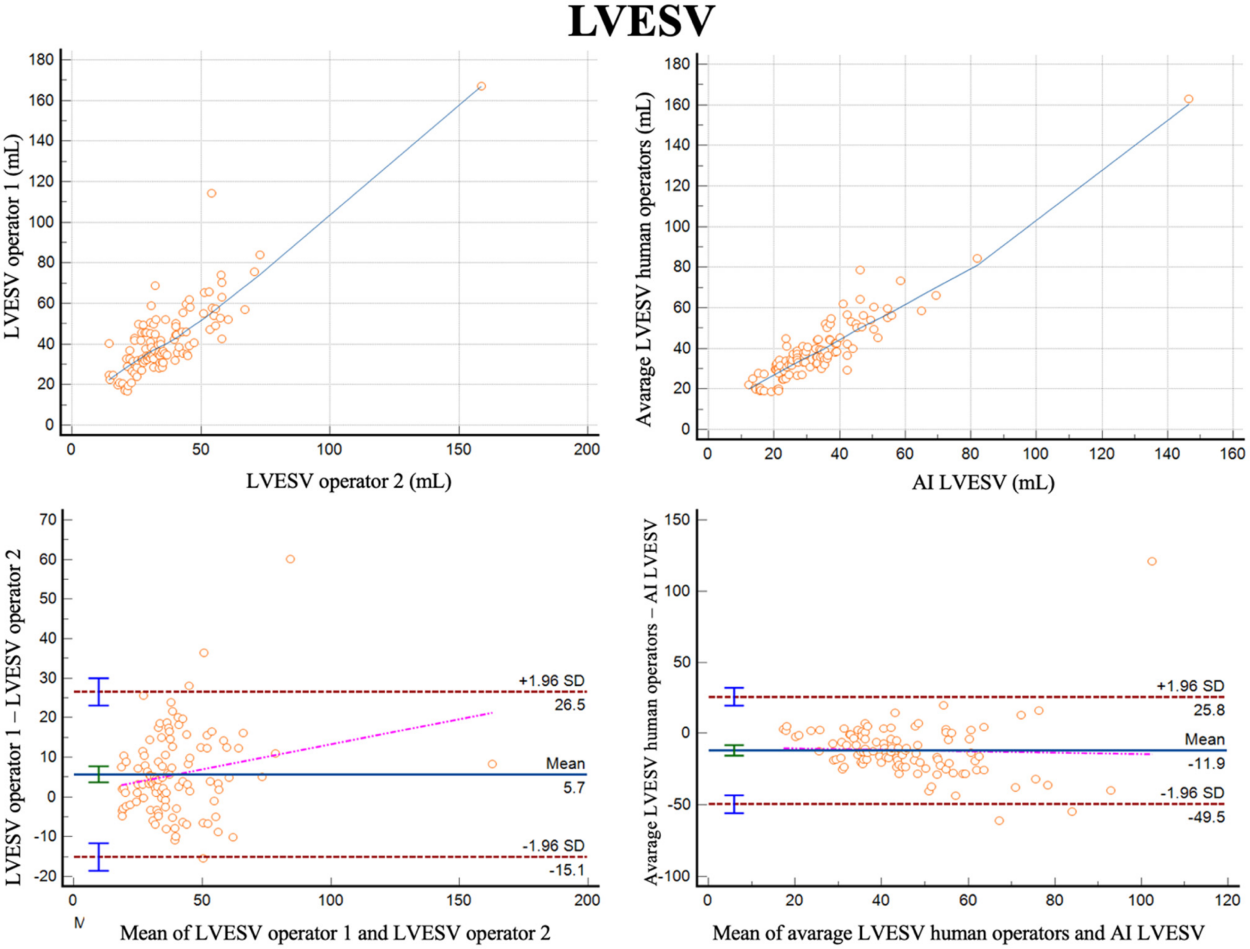
Correlation plots and Bland-Altman plots of left ventricular end systolic volume (LVESV) measures between two human operators (left) and between AI-based measures and the average between the human operators (right).

For measurements of LVESV, the correlation between the two operators was *r* = 0.84 (0.77–0.89, *p* < 0.001), with a reliability of k = 0.91 (Figure 2; Table 3). The average bias was 5.7 ml (LoA ± 20.8 ml). Comparing the average of the operators with AI, the correlation was *r* = 0.92 (0.89–0.95, *p* < 0.001) with a reliability of k = 0.60. The AI algorithm measured higher LVESV, with an average bias of 11.9 ml (LoA 37.6 ml).

**Figure 3:**
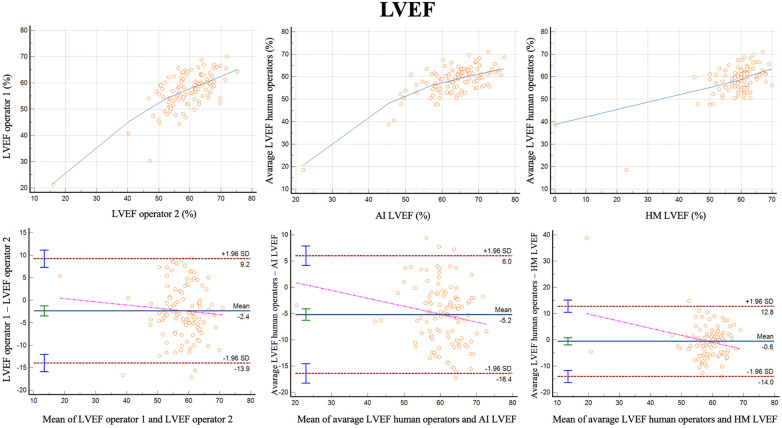
Correlation plots and Bland-Altman plots of left ventricular ejection fraction (LVEF) measures between two human operators (left) and between AI-based measures and the average between the human operators (middle) and between 3D heart model and the average between the human operators (left).

For LVEF the two different operators had a correlation of *r* = 0.68 (0.57–0.77, *p* < 0.001) with a reliability of k = 0.81 (Figure 3; Table 3). The bias was 2.4% (LoA ± 11.5%). Comparing the average of the operators with AI, the correlation was *r* = 0.70 (0.57–0.77, *p* < 0.001) with a reliability of k = 0.82. The bias was −5.2% (LoA ± 11.2%). Additionally, we evaluated the performance of the average of the operators compared to 3DHM technology for the ejection fraction. The correlation was *r* = 0.62 (0.49–0.73, *p* < 0.001) with a reliability of k = 0.76. The bias was −0.6% (LoA ± 13.4%).

**Figure 4:**
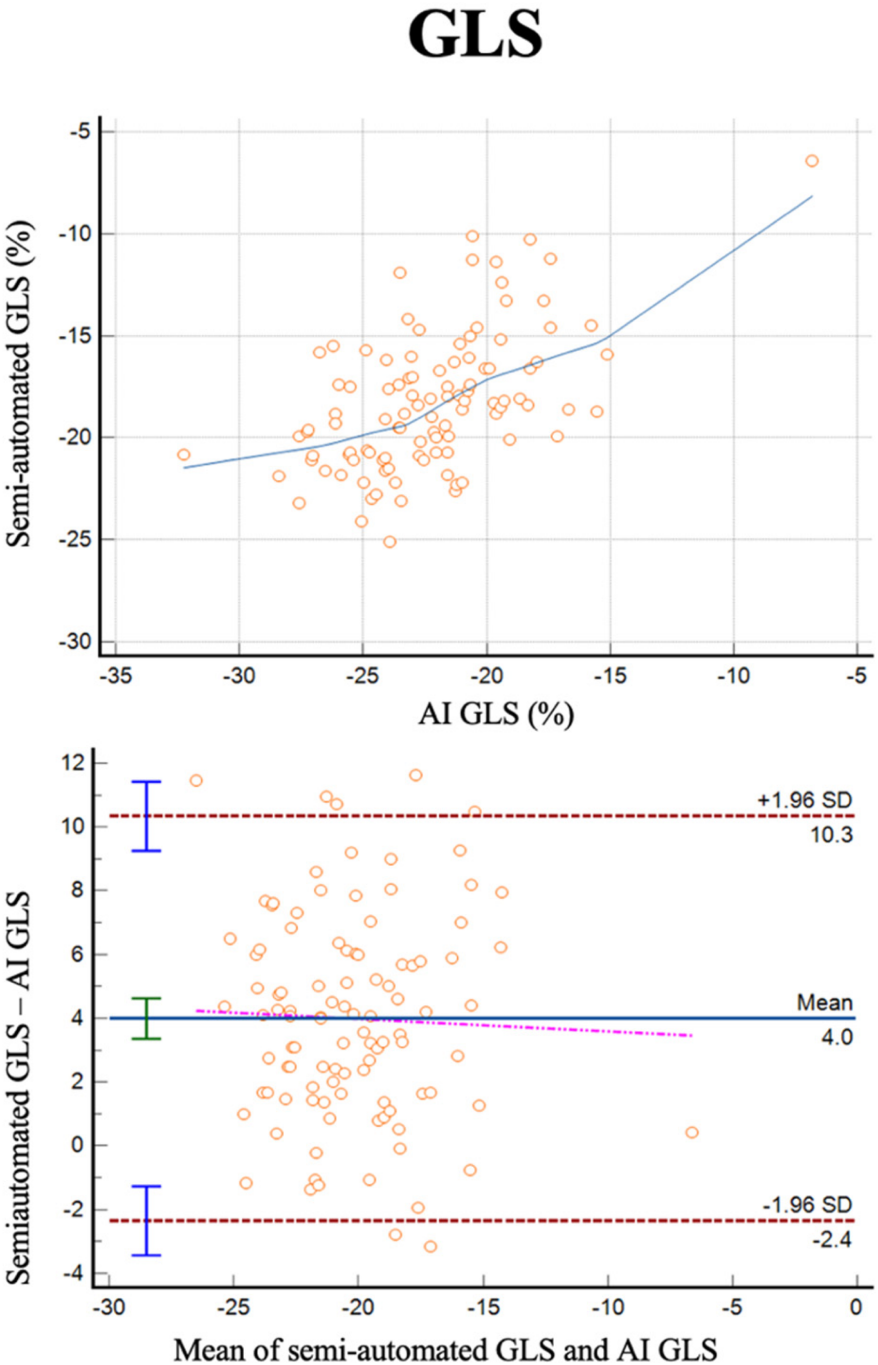
Correlation plots and Bland-Altman plots of left ventricular global longitudinal strain (GLS) measures between AI-based measures and semi-automated measures (autostrain).

GLS was successfully analyzed by human operators and the AI algorithm in 103 subjects (Figure 4). The two methods exhibited a correlation of *r* = 0.55 (0.85–0.92, *p* < 0.0001) with a reliability of k = 0.71 and with and average bias of 4% (LoA ± 6.3%).

Table 3 reports also reports full data for intra-operator variability for LVEDV, LVESV and LVEF.

## Discussion

In this *real-world* study of consecutive subjects who underwent transthoracic echocardiography for various clinical indication, we found good correlations and reliability, and a low bias, for measures of LV volumes and LVEF between human operators and a fully automated AI algorithm. The feasibility of the AI algorithm was high, as all images were successfully analyzed. The 3DHM was able to analyze LVEF in 89% of images, which is in agreement with the feasibility reported in the literature (11), and the accuracy, with human operators as the reference, was inferior to that of the AI model.

AI-based measurements of LVEDV showed superior correlation, agreement, and reliability compared to human operators analyzing identical images. This finding may suggest that AI can mitigate the inherent inter-operator variability that affects the accuracy of conventional echocardiography by standardizing the measurements. The same findings were confirmed for LVESV with respect to correlation, but with a higher bias and a lower reliability for the AI-based measurements. This discrepancy may be attributed to different approaches including myocardial trabeculae in, which become particularly elevated from the pars compacta in end-systole. However, as there were no such differences between measures of LVEDV and LVESV in three larger datasets using the same algorithm, the discrepancies may also be by chance (9).

LVEF is perhaps the most important variable for clinical decision-making. Our data suggest that the agreement, correlation, and reliability of the AI-based algorithm compared to the mean of two operators are nearly identical to those observed between the two operators themselves. This implies that the AI-based algorithm can be considered as reliable and consistent as an experienced operator in measuring LVEF. We also compared the performance of AI-based algorithm with another tool for assessing LVEF, the 3DHM. The 3DHM system exhibited slightly inferior agreement, correlation, and reliability in LVEF measurements compared to the AI-based algorithm when compared to the mean of the two experienced operators. This observation does not justify the added complexity and reduced feasibility associated with automatic 3DHM imaging compared to 2D imaging by the AI-based algorithm. Importantly, 3D measures of LV volumes differ substantially from 2D measures, with 3D and3DHM volumes being closer to LV volumes as measured by cardiac magnetic resonance (11, 12). This may be biased against the 3DHM model, as the reference LVEF was based on 2D-images by human operators.

The correlation between semi-automated and AI-based measures of GLS was modest (*r* = 0.55) and lower than what has previously been reported in larger datasets by the same algorithm (*r* = 0.84 in a real-world dataset and *r* = 0.76 in an echo core lab study of patients with HFpEF) (10). However, as the bias and reliability of the measurements were good, the modest correlation may relate to the narrow range of GLS in this study (majority between −15% and −20%).

Our study has some limitations. This was a retrospective analysis which may have introduced selection bias. We did not validate the findings in an independent cohort, however, the algorithms used have previously been tested in other populations (9, 10). The population studied is rather small with a tight range of LV volumes and EF, mostly within the normal range. The results are representative only for this specific population and can not be generalized to the entire LV volumes and EF range which can be encountered in clinical practice. The images were acquired by equipment from one vendor (Philips Healthcare) and although the AI software is labeled as vendor-independent the findings can not necessarily be extrapolated to other vendors.

## Conclusions

Chamber quantification in echocardiography is crucial for making informed decisions in everyday cardiology practice. Our analysis strongly suggests that an AI-based method for quantifying left ventricle volumes and LVEF can be effectively employed in clinical practice, as it demonstrates good agreement and correlation when compared to assessments made by two experienced human operators.

## Data Availability

The raw data supporting the conclusions of this article will be made available by the authors, without undue reservation.
